# Adjunctive dabigatran therapy improves outcome of experimental left-sided *Staphylococcus aureus* endocarditis

**DOI:** 10.1371/journal.pone.0215333

**Published:** 2019-04-19

**Authors:** Christian J. Lerche, Lars J. Christophersen, Jens Peter Goetze, Pia R. Nielsen, Kim Thomsen, Christian Enevold, Niels Høiby, Peter Ø. Jensen, Henning Bundgaard, Claus Moser

**Affiliations:** 1 Department of Clinical Microbiology, Copenhagen University Hospital Rigshospitalet, Copenhagen, Denmark; 2 Institute of Immunology and Microbiology, University of Copenhagen, Denmark; 3 Department of Clinical Biochemistry, Copenhagen University Hospital Rigshospitalet, Copenhagen, Denmark; 4 Department of Pathology, Zealand University Hospital, Roskilde, Denmark; 5 Institute for Inflammation Research, Department of Rheumatology and Spine Disease, Copenhagen University Hospital Rigshospitalet, Copenhagen, Denmark; 6 Department of Cardiology, Copenhagen University Hospital Rigshospitalet, Copenhagen, Denmark; Institut d'Investigacions Biomediques de Barcelona, SPAIN

## Abstract

**Background:**

*Staphylococcus aureus* is the most frequent and fatal cause of left-sided infective endocarditis (IE). New treatment strategies are needed to improve the outcome. *S*. *aureus* coagulase promotes clot and fibrin formation. We hypothesized that dabigatran, could reduce valve vegetations and inflammation in *S*. *aureus* IE.

**Methods:**

We used a rat model of severe aortic valve *S*. *aureus* IE. All infected animals were randomized to receive adjunctive dabigatran (10 mg/kg b.i.d., *n* = 12) or saline (controls, *n* = 11) in combination with gentamicin. Valve vegetation size, bacterial load, cytokine, cell integrins expression and peripheral platelets and neutrophils were assessed 3 days post-infection.

**Results:**

Adjunctive dabigatran treatment significantly reduced valve vegetation size compared to controls (p< 0.0001). A significant reduction of the bacterial load in aortic valves was seen in dabigatran group compared to controls (p = 0.02), as well as expression of key pro-inflammatory markers keratinocyte-derived chemokine, IL-6, ICAM-1, TIMP-1, L-selectin (p< 0.04). Moreover, the dabigatran group had a 2.5-fold increase of circulating platelets compared to controls and a higher expression of functional and activated platelets (CD62p^+^) unbound to neutrophils.

**Conclusion:**

Adjunctive dabigatran reduced the vegetation size, bacterial load, and inflammation in experimental *S*. *aureus* IE.

## Introduction

*Staphylococcus aureus* is the most frequent cause of infectious endocarditis (IE) [1] and is associated with serious clinical manifestations and fatal course in two out of five patients [2] despite appropriate antibiotic therapy, appropriate management of sepsis and intensive care treatment often combined with cardiac valve surgery. New therapeutic targets enhancing bacterial clearance and dampening the adverse host responses caused by *S*. *aureus* in IE are needed.

The valve vegetation in IE consist of a meshwork of endothelial cells, platelets, fibrin and embedded bacteria [3]. The interactions between host cells and *S*. *aureus* play a pivotal role in the pathogenesis of IE [4], where platelets, leukocytes and endothelial cells modulate the hemostatic activity in response to microorganisms [5,6]. *S*. *aureus* and thrombin are the most potent triggers of platelet activation [7–9] facilitating platelet aggregation, degranulation, release of platelet microbiocidal proteins, adhesive integrins, clotting factors and the release of chemotactic cytokines recruiting neutrophils to the site of infection [10].

Platelets are known for the ability to trigger neutrophil degranulation, referred to as neutrophil extracellular traps (NETs) [6,11–13]. This fundamental innate immune effector function of neutrophils [14] is essential in endovascular infections, but may cause tissue injury [15,16] and promote additional intravascular thrombosis [17–19]. Furthermore, NETs can promote thrombin generation both by platelet-dependent and -independent mechanisms [20] and consequently trigger an exaggerated host response and enhancement of biofilm formation [21].

Coagulase is a key virulence mechanism in *S*. *aureus* IE [22,23] facilitating the establishment and clot formation on cardiac valves resulting in a compromised host response and affecting the efficacy of antibiotics. This common feature of *S*. *aureus* secreting coagulase enhances the progression of valve vegetations and platelet aggregation, which could be a key intervention target disabling the excessive clot formation induced by *S*. *aureus*. Recent studies have shown anti-virulent effect by targeting coagulase in *S*. *aureus* by preventing the complex formation of coagulase-prothrombin complex (staphylothrombin) and thereby inhibits the subsequent facilitated fibrin formation [22,24,25]. Another study has shown beneficial effect of combination of dabigatran and anti-clumping factor in the survival of septic mice [26]. Furthermore, dabigatran was shown to be effective as prophylactic treatment in a low-grade *S*. *aureus* induced model of experimental IE [27].

Dabigatran is a direct thrombin inhibitor (Factor IIa), blocking the conversion of soluble fibrinogen to insoluble fibrin. Direct thrombin inhibitors seem to be a promising adjunctive strategy against intravascular infection caused by *S*. *aureus*. However, no studies have evaluated dabigatran as a therapeutic strategy in already established and severe *S*. *aureus* IE.

We hypothesized that limiting the process of platelet aggregation and fibrin formation involved in *S*. *aureus* infections by dabigatran treatment might be an effective adjunctive therapy in endocarditis. Our previously developed rat model of aortic valve IE was used [28,29].

## Material and methods

Expanded versions of the Methods are presented in the Supplemental material (S1 File).

### Bacterial challenge and growth conditions

*S*. *aureus* (NCTC 8325–4), a laboratory derivative (cured of prophages) of the WT sepsis isolate 8325 was used in the present study [30]. The isolate originates from a patient with *S*. *aureus* IE and expresses key virulence factors involved in IE including coagulase, clumping factors, α and β-hemolysin, protein A. Bacteria were prepared as previously described [28,29].

### Dabigatran treatment

Dabigatran etexilate (Pradaxa, Boehringer Ingelheim, Basel, Switzerland) tablets of 75 mg were dissolved in 9 mL 0.9% saline overnight at 37°C on shaking table (150 rpm) and doses of 10 mg/kg in 300 μL syringe were administrated intraperitoneal (i.p.). Control rats received i.p. injections with 300 μL 0.9% saline.

### Study animals

#### Experimental endocarditis model

High-grade aortic valve catheter induced mechanical lesions were produced in male Wistar rats, weight 225-250g (Janvier Labs, Rennes, France), as previously described [28,29]. Twenty-four hours after induction of the valve lesion rats were inoculated by intravenous injection by 0.5 x 10^7^ CFU of *S*. *aureus* in a tail vein. Catheters were removed immediately before inoculation of the bacteria simulating native valve endocarditis conditions. Sterile thrombotic endocarditis was produced in sham control rats by catheter induced mechanical lesions without injection of bacteria, as previously described [28,29]. Before catheter procedure all animals were anaesthetised with a mixture of Hypnorm (fentanyl 0.315 mg/mL and fluanisone 10 mg/mL), sterile water and midazolam (5 mg/mL) in 1:2:1 dilution. Postoperative all rats received buprenorphine s.c. (0.05 mg/kg) every 8h for 48h post procedure. All rats were maintained in specific-pathogen-free conditions, monitored at least 3 times per day and had free access to water and food ad libitum. Rats reaching an endpoint, suffering or distress were sacrificed with pentobarbital/lidocaine i.p..

#### Intervention groups

All rats with IE were treated with gentamicin 20 mg/kg/day (Hexamycin i.v. solution, Sandoz A/S, Denmark) subcutaneously (s.c.) initiated one day post-infection (DPI) [28,29]. Rats treated with adjunctive dabigatran etexilate received a dose of 10 mg/kg in 300 μL saline solution i.p. at 12 h intervals (b.i.d).

Infected rats with severe *S*. *aureus* IE were randomized into two intervention groups: 1) intervention receiving adjunctive dabigatran (10 mg/kg b.i.d.) and gentamicin (20 mg/kg/day) (dabigatran group, *n =* 12) and 2) intervention receiving saline and gentamicin (saline group, *n* = 11). Rats with catheter-induced valve lesions, but without inoculation of bacteria developed sterile thrombotic endocarditis. These rats were randomized into: 1) sham controls receiving dabigatran (*n* = 6) or 2) sham controls receiving saline only (*n* = 6). The sham control group received the same dose of dabigatran as used for infected rats. All rats were evaluated three days after bacterial inoculation (infected rats) or removal of catheter producing sterile thrombotic endocarditis (sham controls). Intervention groups received two days treatment before evaluation.

#### Quantitative bacteriology

Was performed as previously described [28].

#### Digital planimetry

All infected (*n =* 23) rats and sham controls (*n* = 12) rats were autopsied immediately after lethal i.p. injection of pentobarbital/lidocaine (1 mL of 200/20 mg/mL) and hearts were aseptically dissected for photographic imaging (Sony Cyber-shot DSC-RX100) of the aortic valves. Valve vegetation size (mm^2^) was measured by digital photoplanimetry (ImageJ, v. 1.49m) by a pathologist blinded to treatment regimens.

#### Histopathology

See supplemental material (S1 File).

### Flow cytometry and ROTEM analysis

#### Measurements of platelets, leukocytes and platelet-neutrophil complexes (PNC)

**Reagents**. The following fluorescence-labelled antibodies were used to separate panels. **Panel 1** (neutrophil panel) activated neutrophils CD11b (APC, BD Bioscience, San Jose, CA, US, 562102) and rat granulocytes HIS48 (FITC, BD, 554907). **Panel 2** (neutrophil-platelets complexes); resting platelets CD42d (glycoprotein V) (PerCP/Cy5.5, Nordic Biosite, Copenhagen, Denmark, 148508), activated platelets P-selectin (CD62p) (APC, Nordic Biosite, 148304) and HIS48 (FITC, BD, 554907).

#### For sample preparation and flow cytometer settings

See Supplemental material (S1 File).

**ROTEM analysis**. Fresh frozen plasma samples (-80 °C) were used for rotational thromboelastometry (ROTEM) analysis on ROTEM delta (Tem Systems Inc., Munich, Germany) (S1 File).

**Pharmacokinetics of dabigatran**. Fourteen healthy rats were used for evaluation of pharmacokinetics (PK) in rats injected i.p. with single dose dabigatran (10 mg/kg) (Pradaxa, Boehringer Ingelheim, Basel, Switzerland).

Measurement of cytokine and cell integrins. A 9-plex magnetic beads assay (Bio-Rad, Hercules, CA, US) was used to measure the production of granulocyte–colony-stimulating factor (G-CSF), keratinocyte-derived chemokine (KC, rat analogue to human IL-8), interferon (IFN)-γ, interleukin (IL)-1β, IL-6, IL-10, IL-17A, vascular endothelial growth factor (VEGF), RANTES, (CCL5) (S1 File).

### Ethics statement

All animal experiments were approved by The Animal Ethics Council and The Animal Experiments Inspectorate in Denmark (License no. 2013-15-2934-00952). Animal experiments were conducted according to institutional guidelines at Copenhagen University at Department of Experimental Medicine (accredited by the Association for Assessment and Accreditation of Laboratory Animal Care International (AAALAC) since 2009), Danish laws and regulations and EU Directives and Conventions (https://emed.ku.dk/about/policies/).

### Statistical analysis

All calculations were performed using GraphPad Prism (version 7.2, GraphPad Software, Inc., San Diego, USA). Bacterial tissue densities, cytokines and adhesins were transformed logarithmically (log_10_) and expressed by mean ± standard deviation (SD). Quantitative bacteriology and cytokines were verified by normality test for parametric data and pairwise comparison with Student’s unpaired t-test. For multiple comparisons, one-way and two-way ANOVA Tukey´s multiple comparisons test was applied. Categorical data were analyzed by Chi-square. Flow cytometry were analyzed and calculated with BD FACS Diva software (v.6) and Flow Jo (v. 10). *P* ≤ 0.05 was considered significant.

## Results

### Pharmacokinetics of dabigatran

**Fig 1:**
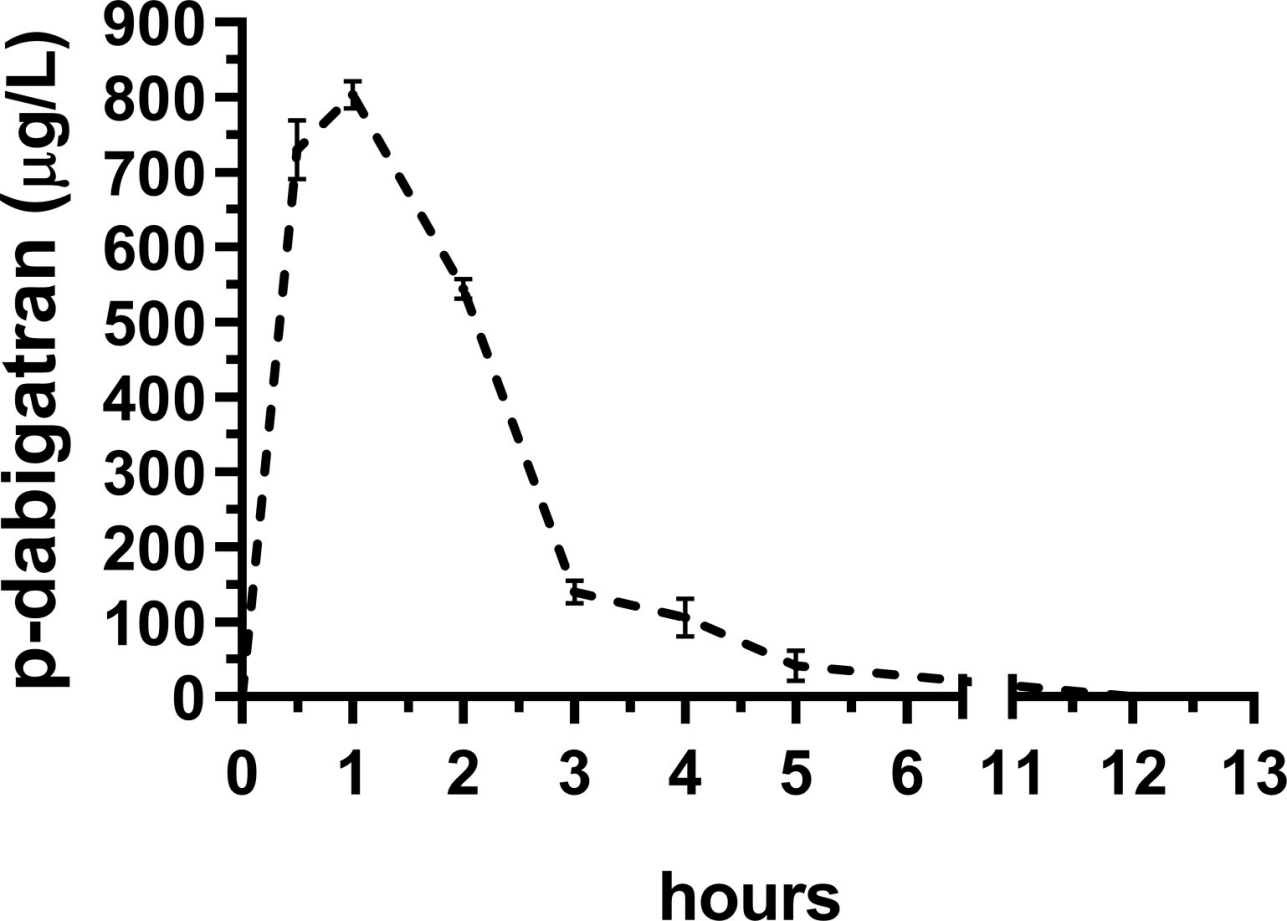
Dabigatran pharmacokinetics (PK). PK of dabigatran etexilate administrated by one dose of 10 mg/kg intraperitoneal. Plasma dabigatran (p-dabigatran) were measured in two healthy rats at each time point (*n* = 14). A peak p-dabigatran concentration was seen after 1 hour (~800 μg/L) and though value was seen at 5 hours (40 μg/L).

Dabigatran peak plasma concentrations (C_max_, ~800 μg/L) were reached within one-hour post injection of the prodrug, indicating rapid absorption and conversion to active dabigatran. Dabigatran trough plasma concentration was measured after 5 hours (C_min_, 40 μg/L). There was no measurable residual dabigatran in the plasma after 12 hours (**Fig 1**).

### Effect of dabigatran on valve vegetation size

**Fig 2:**
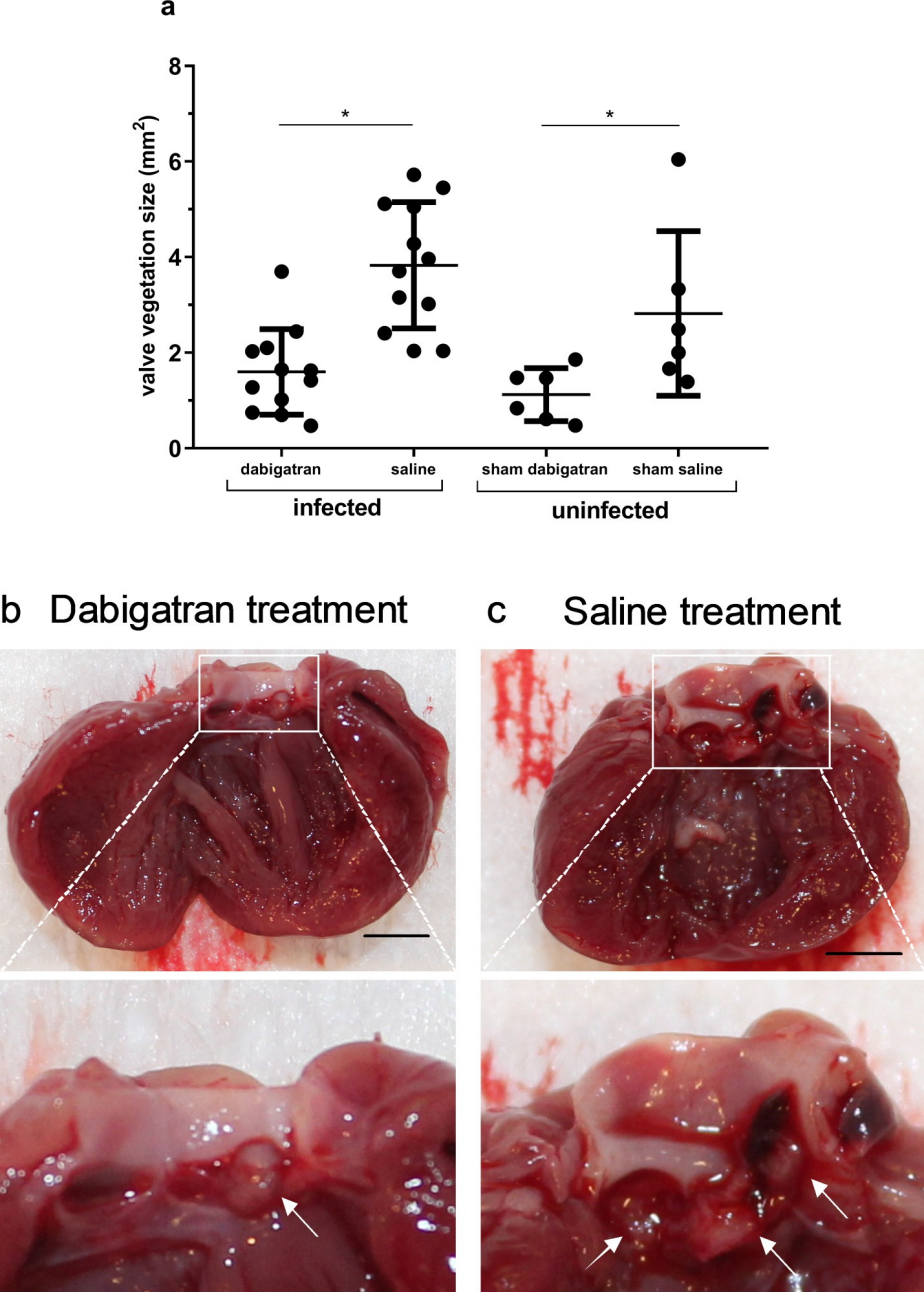
Dabigatran effect on valve vegetation size. A significant reduction in valve vegetation size was seen for dabigatran treated (10 mg/kg/b.i.d) compared to saline treated rats with *S*. *aureus* infective endocarditis (infected) evaluated 3 days post inoculation (**Fig 2A**). Both infected groups received gentamicin 20 mg/kg/day (s.c.). A significant reduction of the valve vegetation size was seen in sham control (uninfected) with sterile thrombotic endocarditis in the dabigatran treated (10 mg/kg/b.i.d) group compared to saline controls evaluated 3 days post removal of catheter (**Fig 2A**). Representative macroscopic images of valve vegetations from one rat treated by dabigatran (**Fig 2A**) and saline (**Fig 2C**), arrows indicating the valve vegetations located at the aortic leaflets. Black scale bar indicates 3 mm. Horizontal lines represent means ± standard derivation. * indicate p < 0.05 by unpaired t-test.

The aortic valve vegetations were significantly reduced in size in dabigatran compared to saline treated animals (1.6 ± 0.9 vs 3.8 ± 1.3 mm^2^, p < 0.0001) (**Fig 2A**). A similar difference was seen in uninfected sham controls receiving dabigatran vs. saline (1.1 ± 0.6 vs. 2.8 ± 1.7 mm^2^, p = 0.04) (**Fig 2A**). Representative macroscopic images of valve vegetations from dabigatran and saline treated group are illustrated in **Fig 2B** and 2C, respectively.

### Effects of dabigatran on the bacterial load in infected valves

**Fig 3:**
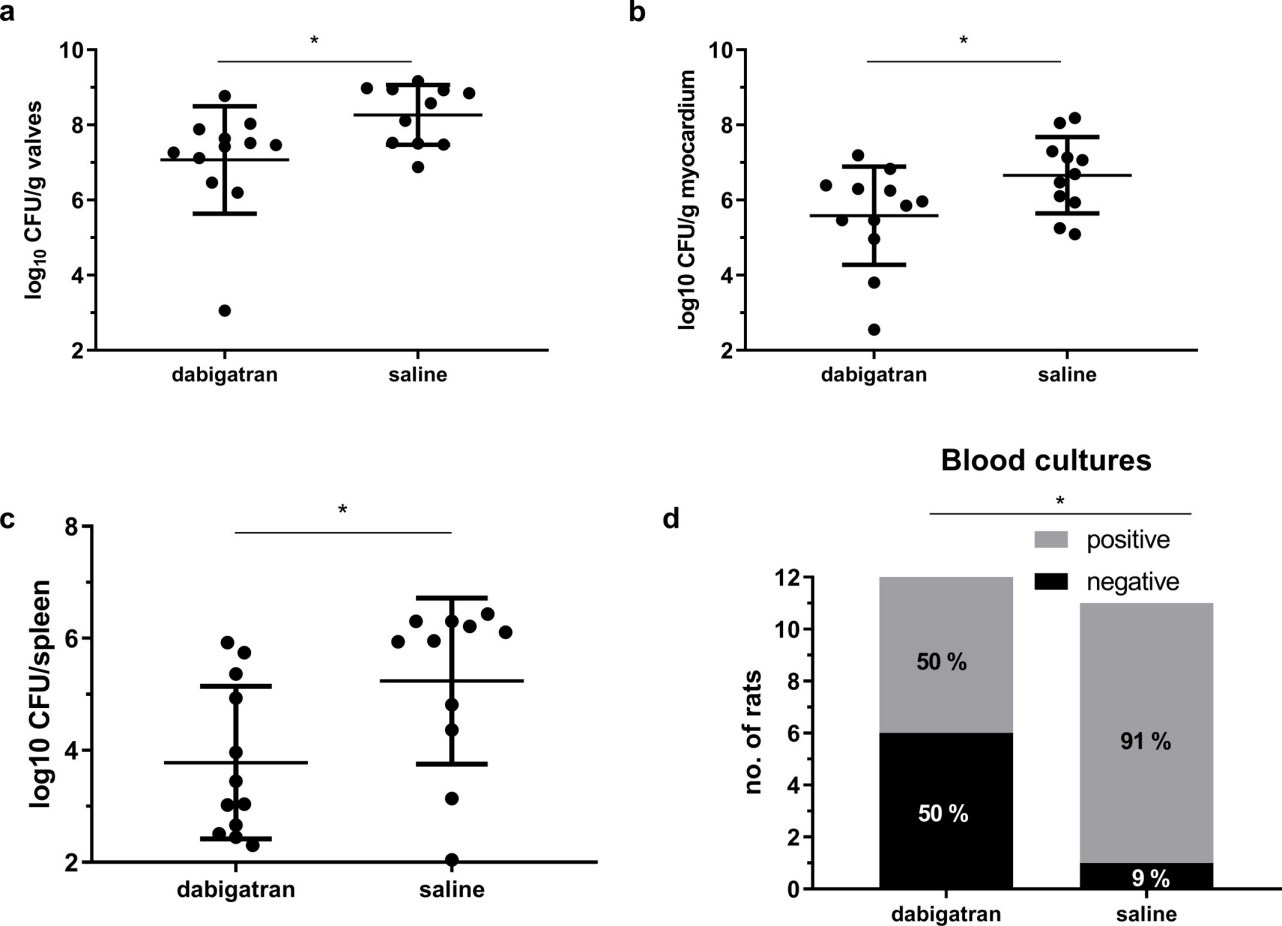
Dabigatran effect on bacterial load. A significant reduction in the bacterial load were seen in valves **(A)**, myocardium **(B)** and spleen **(C)** in dabigatran (10 mg/kg/b.i.d) compared to saline treated group. A significant reduction of positive blood cultures in the dabigatran compared to saline group **(D)** was seen. Horizontal lines represent means ± standard derivation. * indicate p < 0.05 by unpaired t-test **(A-C)** and chi-square test **(D)**.

A significant reduction of ~1 log colony-forming units (CFU) was seen in the valve vegetations in the group receiving dabigatran compared to the group receiving saline (7.01 ± 1.4, *n* = 12 vs. 8.03 ± 0.8, *n =* 11, p = 0.02) (**Fig 3A**).

### Effect of dabigatran on the bacterial load in myocardium, spleen, and blood

Similar reductions were seen in myocardium (5.58 ± 1.3 vs. 6.66 ± 1.0, p = 0.04) (**Fig 3B**) and spleen (3.78 ± 1.4 vs. 5.24 ± 6.2, p = 0.02) (**Fig 3C**) comparing the two intervention groups. Rats receiving dabigatran treatment had 50% negative blood cultures compared to 9% in the saline group (p = 0.03) (**Fig 3D**). In kidneys, tissue negative cultures were seen in 33% (4/12) of dabigatran treated compared to 9% (1/11) in the saline group (p = 0.31) (data not shown). All the uninfected sham controls were culture negative from blood and aortic valves.

Two animals perished during the treatment course and were excluded from the evaluation. One, in the dabigatran group, due to peritoneal fecal co-infection (possible cage acquired infection) and the other in the saline group due to severe progression of IE with intracerebral affection with limb paresis.

### Effect of dabigatran on cytokine and integrin levels in affected valves

**Fig 4:**
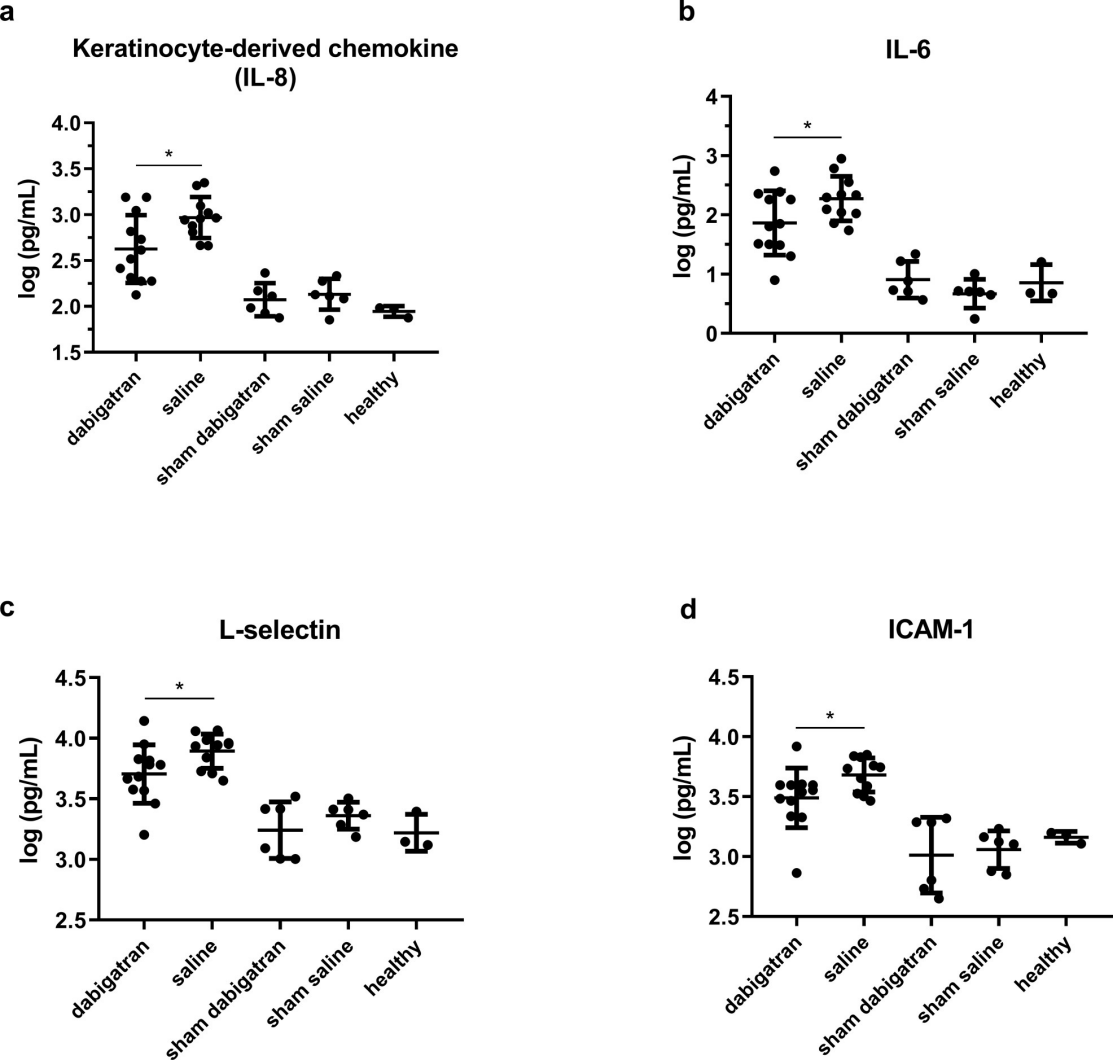
Dabigatran effect on cytokines and integrins in valves. A significant reduction in key inflammatory markers as keratinocyte-derived chemokine (rodent homolog to human interleukin 8 (IL-8)) (A), IL-6 (B), L-selectin (C), ICAM (D), in infected rats was seen in dabigatran compared to saline treated.

All cytokine and adhesin markers were elevated in rats with IE compared to rats with sterile thrombotic endocarditis, indicating a highly mobilized and active host response in valves colonized with *S*. *aureus* (**S1 Table**). Keratinocyte-derived chemokine (KC) (analogue to human IL-8) is the most sensitive marker for inflammation in valve tissue of IE, followed by IL-6 and IL-1β [28]. An almost 2-fold reduction of KC (**Fig 4A**) and IL-6 (**Fig 4B**) were seen in the dabigatran compared to the saline group (594 ± 523 vs. 1057 ± 590 pg/mL, p < 0.01 and 137 ± 155 vs. 269 ± 259 pg/mL, p < 0.05, respectively). IL-1β showed a non-statistical significant 1.4-fold reduction in the dabigatran treated group (p = 0.09) and RANTES 1.4-fold increase (p = 0.09) (**S1 Table**). Ratio between pro-inflammatory and anti-inflammatory expression by KC and IL-10, KC and RANTES showed significant reduction in the dabigatran group compared to the saline group (p = 0.03 and p = 0.02, respectively). A significant reduction of RANTES were seen in sham controls receiving dabigatran compared to saline treated sham controls (p < 0.03) (**S1 Table**).

Important cell surface adhesins and glycoproteins expressed by platelets, leukocytes and endothelial cells were quantified showing significant reduction of L-selectin and ICAM-1 in the dabigatran treated compared to saline treated (5802 ± 3215 vs. 8189 ± 2443 pg/mL and 3518 ± 1808 vs. 8189 ± 2443 pg/mL, p < 0.04, respectively) (**Fig 4C and 4D**). Interestingly, tissue metallopeptidase inhibitor 1 (TIMP-1) expressed by endothelial cells and platelets, an important reactivity marker for these cells, revealed a significant reduction in the dabigatran treated compared to saline treated rats (8095 ± 11186 vs. 14619 ± 9026, p < 0.02) (**S1 Table**). Importantly, uninfected sham controls were observed to have a significant reduction of P-selectin in dabigatran treated compared to saline treated, indicating reduced platelet aggregation in valves (489 ± 642 vs. 2686 ± 548 pg/mL, p < 0.0001) (**S1 Table**), however for infected rats only a non-significant reduction in P-selectin was seen in dabigatran treated compared to saline treated group (**S1 Table**).

No difference was seen between the two uninfected dabigatran or saline treated sham control groups. Concerning von Willebrand factor (vWF), thrombin-antithrombin complexes (TAT) and tissue factor (TF) no differences was found between the two intervention groups. However, TAT was significantly increased in dabigatran treated sham compared to saline sham controls (p = 0.05) (**S1 Table**).

### Neutrophils, platelets and platelet-neutrophil-complex in *S*. *aureus* IE

To investigate the interaction between platelets and neutrophils in the circulation during IE, we measured the complex binding between platelets and neutrophils. Platelet-neutrophil complex formation was high in both intervention groups (mean fluorescence index (MFI) ~4200), but no difference was observed (**S2A Fig**).

**Fig 5:**
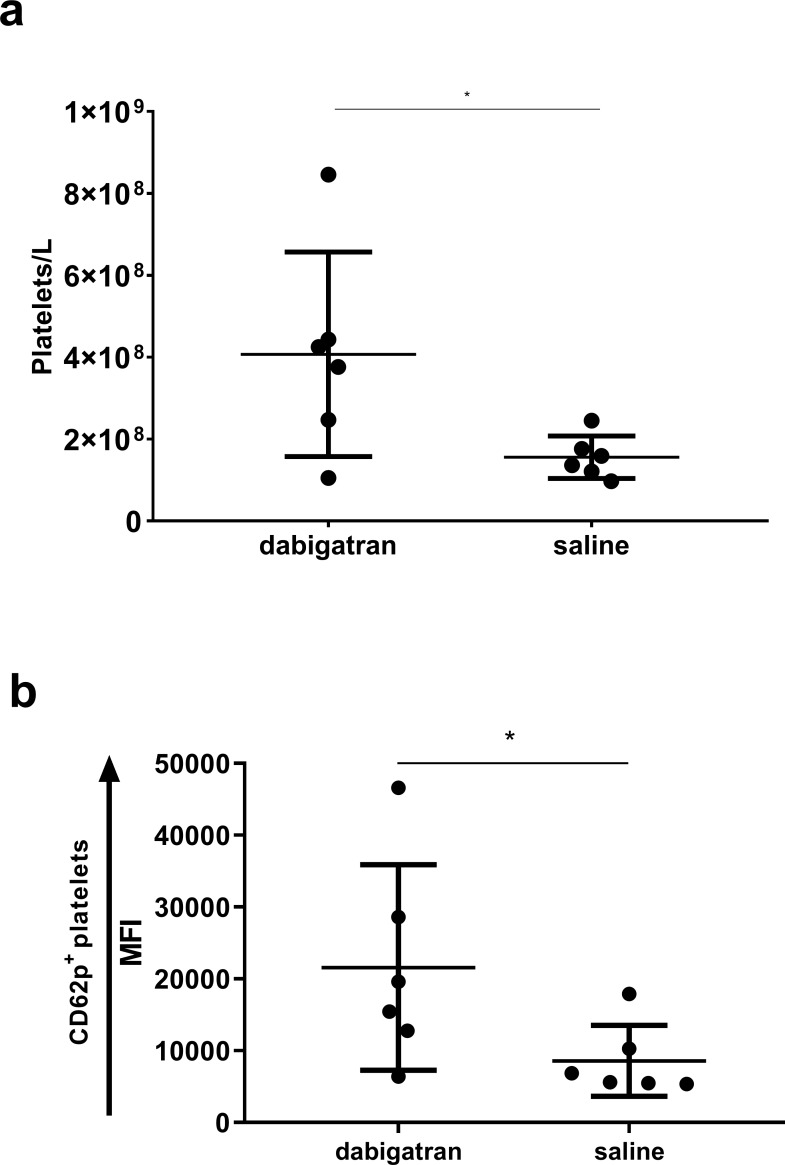
Flow cytometric analysis of platelets. Platelets count **(A),** unbound activated platelets expressed by mean fluorescence index (MFI) (CD62p^+^) (B), Horizontal lines represent means ± standard derivation. * indicate p < 0.05.

Interestingly however, the infected group receiving adjunctive dabigatran had a significant 2.5-fold increase of circulating platelets unbound to leukocytes compared to the saline group (mean of 4.0 vs. 1.6 x 10^8^ platelets/L, respectively), indicating a higher turnover of platelets in the latter group (**Fig 5A**). Moreover, the number of activated platelets (expressing CD62p) unbound to leukocytes was significantly higher in the dabigatran vs. saline group (MFI, 21569 ± 14315 vs MFI 8576 ± 4926, p = 0.04), indicating a higher number of functional and activated platelets in the circulation (**Fig 5B**).

Neutrophils and total leukocyte count (TLC) measured by flow cytometry revealed a statistical non-significant reduction in the dabigatran treated group of neutrophils (mean 4.39 vs 8.77 x 10^9^ neutrophils/L, p = 0.20) and TLC (mean 7.11 x 10^8^ vs. 1.25 x 10^9^ TLC/L, p = 0.18) respectively (**S3B and S3C Fig**).

### Rotational thromboelastometry (ROTEM) analysis in rats with *S*. *aureus* IE

No significant difference was found between the two infected intervention groups in regard to clotting time (CT), clot formation time (CFT), alpha angle or maximum clot formation (MCF). Showing comparable hemostatic functionality of the intrinsic and extrinsic pathways, no increased bleeding time was observed in either groups (**S2 Table)**.

### Histopathologic characteristics of tissue specific inflammation in *S*. *aureus* IE

For results see **S3 Table** and illustrative figures (**S1 and S2 Figs)** in supplemental material.

### Interaction between *S*. *aureus* and dabigatran in a plasma assay

See supplemental material (**S4 Fig**).

## Discussion

In the present study of experimental aortic valve *S*. *aureus* IE, we demonstrated that adjunctive dabigatran therapy combined with gentamicin reduces the valve vegetation size, bacterial load on the valves, in the myocardium, spleen and fewer persistently positive blood cultures of dabigatran treated rats.

Furthermore, dabigatran reduced the key pro-inflammatory cytokines KC (IL-8), IL-6 and IL-1β [28,29], cell integrins and metalloproteases L-selectin, ICAM-1 and TIMP-1 in valve vegetations. The levels of IL-8, IL-6 and IL-1β is primarily released by activated platelets, indicating reduced platelet aggregation and neutrophil shedding of L-selectin upon activation, tethering and adhesion [31] of the inflamed endothelium. The reduced TIMP-1 expression correlated to the reactivity and number of platelets within the vegetation [32]. P-selectin was significantly reduced in uninfected sham controls receiving dabigatran compared to saline sham controls, indicating reduced aggregation of platelets in sterile thrombotic vegetations. However, this effect could not be identified in the infected rats, probably due to the severe inflammation and high bacterial burden of both groups at the time of evaluation.

Interestingly, RANTES levels were elevated in infected valves and inversely correlated to pro-inflammatory cytokines, showing a decreased ratio score of KC/RANTES (pro-inflammatory/anti-inflammatory) in the dabigatran treated group compared to the saline treated group (p<0.02). This observation may be indicative of the recruitment of monocytes (removing debris and promoting tissue healing), increased monocyte-platelet crosstalk stimulating anti-inflammatory signals (e.g. IL-10 production) [33] and signaling toward an earlier activation of the adaptive immune response [34]. This speculation is supported by the fact that the observation adheres to the infection and not the sterile vegetations since the RANTES level is comparable to the background level of healthy controls. The anti-inflammatory effect of dabigatran was also seen by reduced ratio score of KC/IL-10.

Between the two intervention groups there were no difference in platelet-neutrophil complexes. Although, this is in accordance with observations in a sepsis model of streptococci [35], we observed significant difference in the platelet turnover between the two groups, where the dabigatran treated group had a higher platelet count and a higher number of functional activated platelets compared to saline treated. Reduction in platelets is a well-known biomarker for disease severity and our observations highlight the anti-virulent effect of dabigatran in our *S*. *aureus* IE model. Patients with bacteremia has a high number of platelet-leukocyte complexes compared to healthy controls. However, in patients with complicated sepsis with organ failure, the number of platelet-leukocyte complexes has shown to be decreased, probably a consequence of a high turnover of platelets sequestrating as micro-thrombosis causing organ ischemia, tissue and vessel damage [36].

Various disease models and *in vitro* studies have shown the important aspect between platelets and neutrophils in intravascular infections. Platelets, neutrophils and the formation of complexes between the two is an important effector function of the innate immunity in the battle against *S*. *aureus* in endovascular infections. However, cross-talk between the coagulation system and innate immunity promotes coagulation [4,37], induces biofilm formation in IE [21] and upregulates the pro-inflammatory cytokine production [28,29,38] for recruitment of neutrophils promoting additional inflammation by degranulation (NET release) in the valves [39,40].

Dabigatran shows promising signs in a potential strategy to dampen the exaggerated host response between platelets and neutrophils triggered by *S*. *aureus*. In vitro, it has been shown that dabigatran in high concentrations can reduce platelet activation, and in low concentrations inhibit thrombin induced platelet aggregation [41] with combined stimulation by platelet agonists. Another way of attenuating bacteria-induced inflammation has been demonstrated in whole blood models [42], however in such a setting, it is important to recognize the direct anti-virulent effect of direct thrombin inhibitors towards *S*. *aureus*.

Furthermore, dabigatran has shown to promote the fibrinolytic activity altering the clot structure [43], which might contribute to reduced resistance against the host response and antimicrobial agents. Dabigatran could have similar effects, as shown by treatment with recombinant protein A2 (fragment of vWF) in lipopolysaccharide-induced DIC murine model, by reducing fibrin-rich microthrombi dissemination [44].

The hemostatic function and clot formation time were comparable between the two intervention groups, indicating no accumulation of dabigatran and no excess coagulation times of dabigatran treated rats, although we observed high peak concentration in healthy controls with the chosen treatment dose. Coagulopathy in sepsis and IE is a dynamic process; sequential measurements are needed to evaluate the *in vivo* effect of dabigatran in *S*. *aureus* IE, although beyond the scope of this study.

To assess the safety of adjunctive dabigatran, tissue samples were evaluated by histopathology. Importantly no increased hemorrhagic tissue damage in the myocardium, spleen and kidney was observed in dabigatran treated. Furthermore, a trend toward decreased inflammation and kidney necrosis of dabigatran treated was observed, which may be explained by reduced microthrombi sequestering in the kidneys. No additional inflammatory differences were seen between the intervention groups, but this could probably be explained by the low number of animals in the assessment.

Our blood cultures findings are in correlation with the first prospective randomized study comparing dabigatran treatment to standard thromboprophylaxis of patients with *S*. *aureus* bacteremia [45], which showed acceptable and similar rates of bleeding events in 94 enrolled patients. The study [45] also observed a trend of less persistently blood cultures in dabigatran treated patients as we observed in our study, indicating augmented effects of gentamicin and the host response.

Coagulase activity is expressed in all *S*. *aureus* making this pathogen specific target a potential strategy, as presented in this study, by blocking the formation of staphylothrombin complexes [24,45]. This direct effect is likely not applicable for other pathogens, but future studies will need to clarify the role of dabigatran in Gram-positive bacteria e.g. streptococcal and enterococcal IE.

Multiple preclinical studies of IE have shown the benefit of antiplatelet therapy [46,47] and combination of ticlopidine and aspirin to prevent IE in *S*. *aureus* and *Streptococcus gordonii* [27]. Furthermore, dabigatran was protective in 75% of rats infected by *S*. *aureus*, however failed to be protective against *S*. *gordonii* [27], highlighting dabigatran direct effect toward *S*. *aureus* also demonstrated in our study. The benefit of therapeutic anticoagulation in native IE has never been demonstrated efficient in a clinical setting. Current guidelines from ESC [48] and American Heart Association [49] does not recommend anticoagulation, but we lack well-designed clinical randomized studies in IE to out rule the use of anticoagulant therapy in native endocarditis.

The present study is first proof-of-concept of dabigatran as beneficial adjuvant treatment of established severe *S*. *aureus* aortic valve endocarditis. Our study illustrates that by targeting platelet activation pathways, we can reduce the platelet and neutrophil induced inflammation and biofilm formation on cardiac valves promoted by *S*. *aureus*. Left-sided endocarditis with *S*. *aureus* is an infectious disease with an unchanged and undesirable high frequency of severe complications and fatality rate. Therefore, new treatment options to improve the outcome are needed. Such new treatment strategies are extremely challenging to develop and test directly in clinical randomized trials of IE, currently with only two IE studies performed [50,51].

The present rat model of severe left-sided aortic *S*. *aureus* endocarditis is suitable for preclinical research on potential beneficial treatments of patients with IE [28,29]. In our model, we mimic the cause of progressive *S*. *aureus* IE and hypothesized improvement of dabigatran as adjunctive treatment in the early management of *S*. *aureus* IE.

In conclusion, the present study demonstrates that dabigatran augments the antibiotic efficacy by reducing the valve vegetation size, platelet aggregation, bacterial load, inflammation, and dissemination in an experimental model of native *S*. *aureus* aortic valve endocarditis.

## Supporting information

S1 Fig Histopathological representative illustrations of catheter induced *Staphylococcus aureus* (*S*. *aureus*) infective endocarditis (IE). **(A)** Valve vegetation of severe *S*. *aureus* IE by hematoxylin and eosin stain (H&E) (Magnification x10). **(B)** fibrin (red) sequestering *S*. *aureus* in valve vegetations (x10) Martius Scarlet Blue (MSB). **C)** illustrating aortic root abscess in myocardium (x2.5) with **(D)** severe neutrophil inflammation and inflamed valve (x20). **e)** spleen with hyperplasia of white pulp with (x20) **(F)** CD61 positive megakaryocytes (x20) **(G)** showing two large areas of necrosis in the renal cortex, one at each pole of the kidney, the biggest marked by a dark square (overview), (H) enlarged picture of the area marked by the dark square in picture (G) showing the characteristic infarction with tissue necrosis in the kidney (x2.5).
(TIF)

S2 Fig Histopathological illustrations from rats in the saline group. (**A**) Severe neutrophil infiltration of the aortic valve and subendothelial (hematoxylin and eosin (H&E), magnification x20) and (B) counterstained with Martius Scarlet Blue (MSB) where the collagen is strongly blue but no stains for fresh (yellow) or mature (red) fibrin. (C) Inflamed spleen (H&E, magnification x40) and (D) counterstained with myeloperoxidase (MPO), magnification x40) displaying a high intracellular expression of MPO in the neutrophils.
(TIF)

S3 Fig Flow cytometry of whole blood. Platelet-neutrophil complexes (PNC) **(A)**, neutrophils **(B)** and total leukocyte count (TLC) **(C)** are shown for the two intervention groups. Horizontal lines represent means ± standard derivation. * indicate p < 0.05. n.s., non-significant.
(TIF)

S4 Fig Dabigatran inhibits growth of *S*. *aureus* in plasma. Growth curve of *S*. *aureus* incubated in plasma from rats at different dabigatran concentrations (25–730 μg/L) showing a delayed growth rate of *S*. *aureus* at high plasma dabigatran concentrations (544 and 730 μg/L), indicating a direct anti-*S*. *aureus* effect of dabigatran in plasma. P < 0.0001 at plasma dabigatran concentrations at 730 and 544 μg/L from 1–8 hours compared to < 25 μg/L plasma dabigatran. Experiments were performed in duplicates. Symbols and error bars indicating mean ± SEM.
(TIF)

S1 Table Cytokines and adhesion molecules expression in aortic valve endocarditis. (DOCX)

S2 Table Results of ROTEM performed in plasma samples. (DOCX)

S3 Table Histopathological assessment. (DOCX)

S1 File Extended Material, Method and Results section. (PDF)
