# Supplementary figures and images for: Adjunctive dabigatran therapy improves outcome of experimental left-sided Staphylococcus aureus endocarditis
Source: PLoS One. 2019 Apr 19;14(4):e0215333. doi: 10.1371/journal.pone.0215333 (PMC6474597; doi:10.1371/journal.pone.0215333)

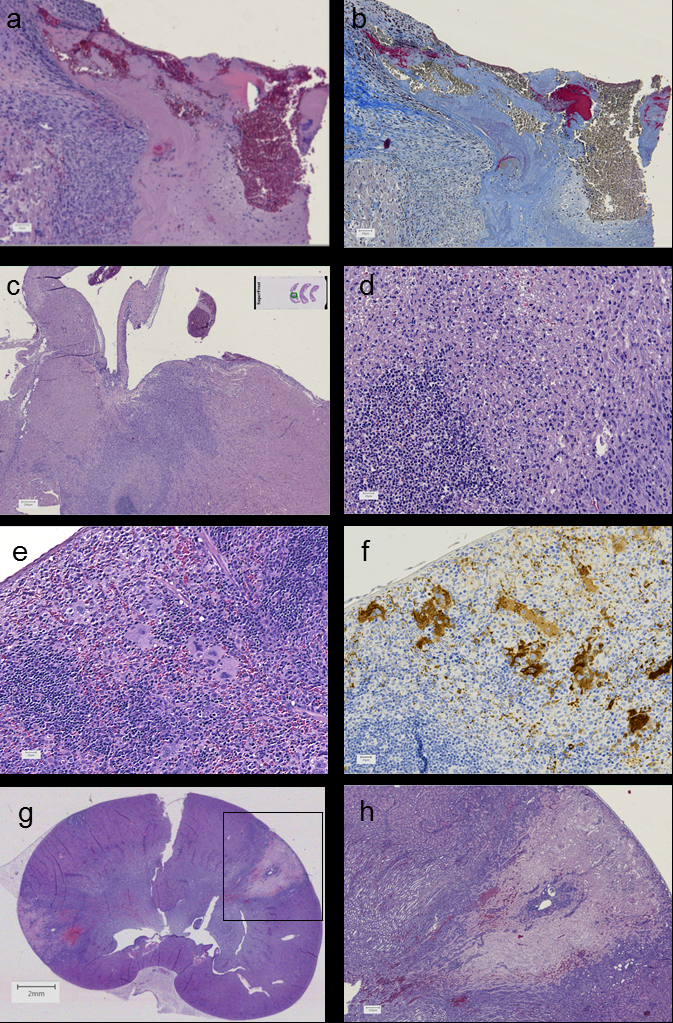

Supplement: S1 Fig — (A) Valve vegetation of severe S. aureus IE by hematoxylin and eosin stain (H&E) (Magnification x10). (B) fibrin (red) sequestering S. aureus in valve vegetations (x10) Martius Scarlet Blue (MSB). C) illustrating aortic root abscess in myocardium (x2.5) with (D) severe neutrophil inflammation and inflamed valve (x20). e) spleen with hyperplasia of white pulp with (x20) (F) CD61 positive megakaryocytes (x20) (G) showing two large areas of necrosis in the renal cortex, one at each pole of the kidney, the biggest marked by a dark square (overview), (H) enlarged picture of the area marked by the dark square in picture (G) showing the characteristic infarction with tissue necrosis in the kidney (x2.5). (TIF) [file pone.0215333.s001.tif]

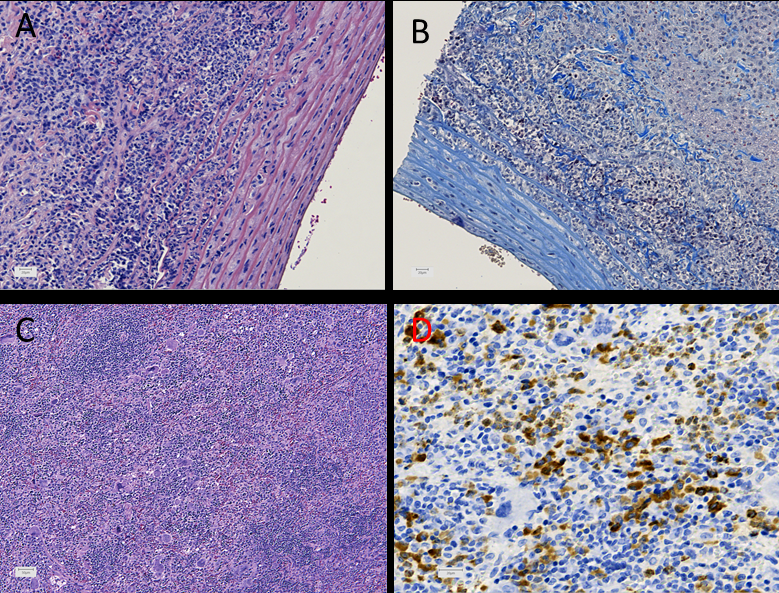

Supplement: S2 Fig — (A) Severe neutrophil infiltration of the aortic valve and subendothelial (hematoxylin and eosin (H&E), magnification x20) and (B) counterstained with Martius Scarlet Blue (MSB) where the collagen is strongly blue but no stains for fresh (yellow) or mature (red) fibrin. (C) Inflamed spleen (H&E, magnification x40) and (D) counterstained with myeloperoxidase (MPO), magnification x40) displaying a high intracellular expression of MPO in the neutrophils. (TIF) [file pone.0215333.s002.tif]

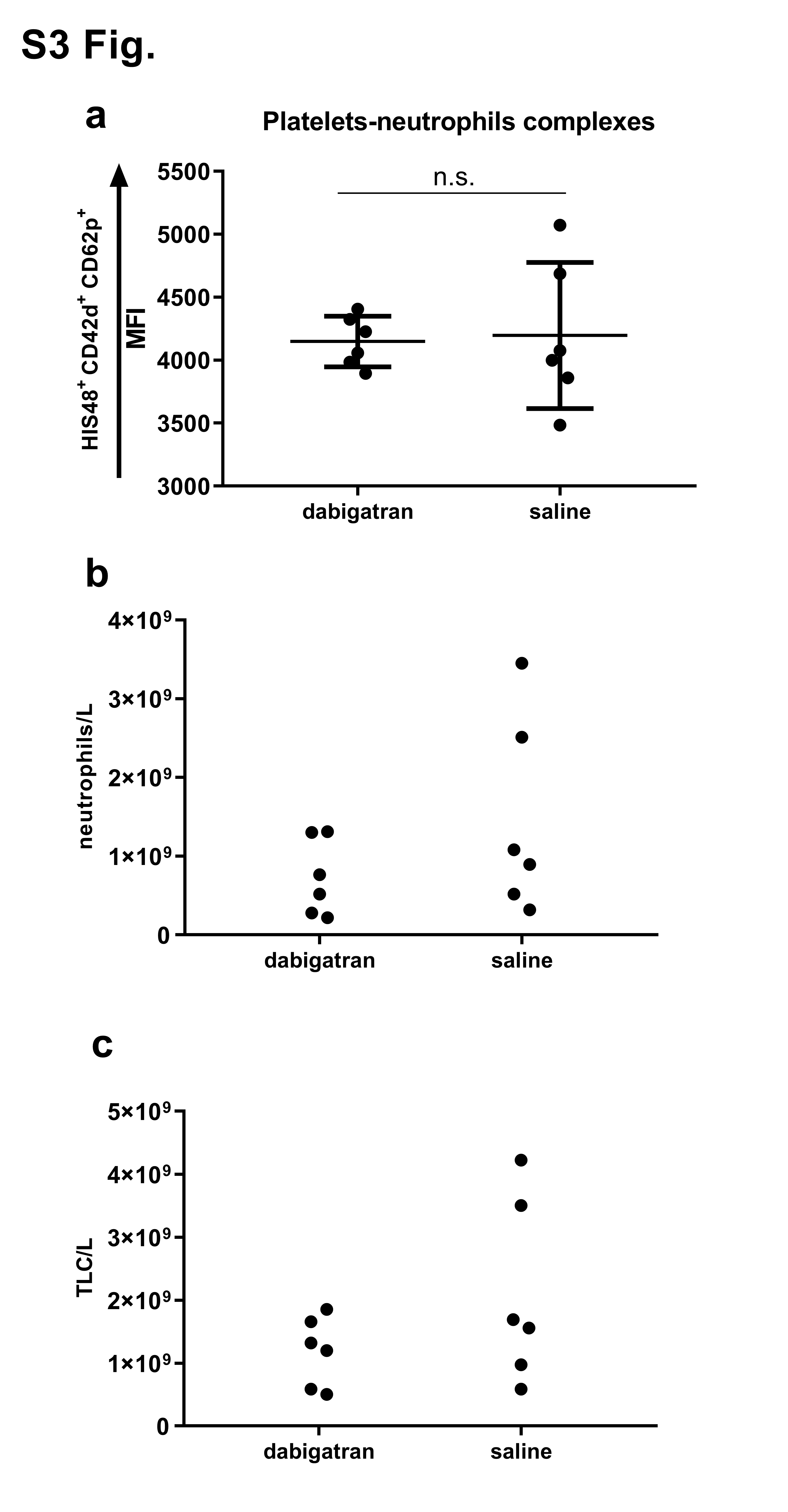

Supplement: S3 Fig — Platelet-neutrophil complexes (PNC) (A), neutrophils (B) and total leukocyte count (TLC) (C) are shown for the two intervention groups. Horizontal lines represent means ± standard derivation. * indicate p < 0.05. n.s., non-significant. (TIF) [file pone.0215333.s003.tif]

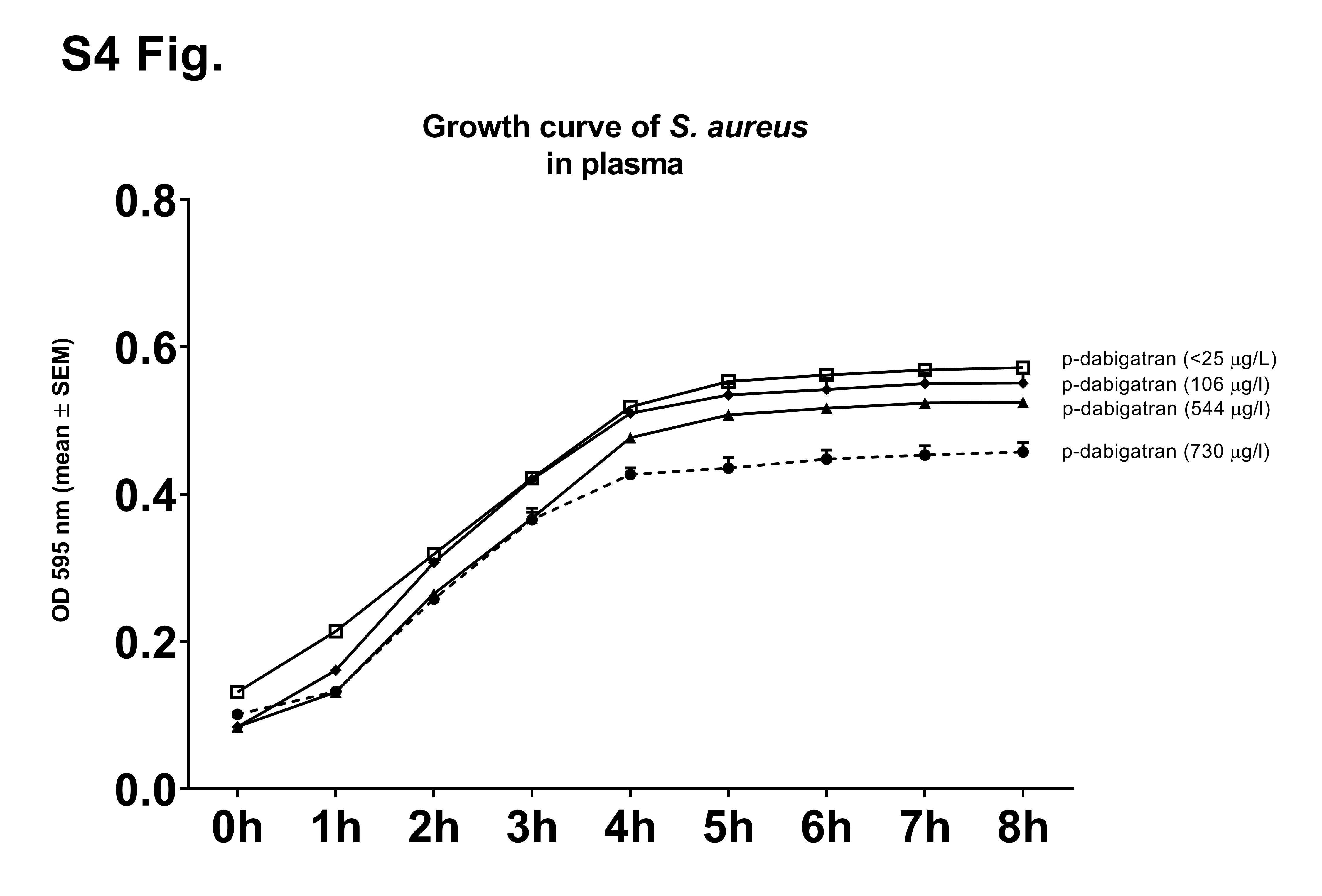

Supplement: S4 Fig — Growth curve of S. aureus incubated in plasma from rats at different dabigatran concentrations (25–730 μg/L) showing a delayed growth rate of S. aureus at high plasma dabigatran concentrations (544 and 730 μg/L), indicating a direct anti-S. aureus effect of dabigatran in plasma. P < 0.0001 at plasma dabigatran concentrations at 730 and 544 μg/L from 1–8 hours compared to < 25 μg/L plasma dabigatran. Experiments were performed in duplicates. Symbols and error bars indicating mean ± SEM. (TIF) [file pone.0215333.s004.tif]
