# Supplementary material for: Adjunctive dabigatran therapy improves outcome of experimental left-sided Staphylococcus aureus endocarditis
Source: PLoS One. 2019 Apr 19;14(4):e0215333. doi: 10.1371/journal.pone.0215333 (PMC6474597; doi:10.1371/journal.pone.0215333)
Supplement: S1 Table — (DOCX) [file pone.0215333.s005.docx]

| S1 Table. Cytokines and adhesion molecules expression in aortic valve endocarditis | | | | | | | | | | | |
| --- | --- | --- | --- | --- | --- | --- | --- | --- | --- | --- | --- |
|  | ***S. aureus* IE** | | |  | **Sterile endocarditis** | | |  |  | |  |
| Cytokines and adhesion molecules  (glycoproteins) | **Dabigatran**  (*n* = 12)  mean ± SD  (pg/mL) | **Saline**  (*n* = 11)  mean ± SD  (pg/mL) | |  | **Dabigatran**  **sham**  (*n* = 6)  mean ± SD  (pg/mL) | **Saline**  **sham**  (*n* = 6)  mean ± SD  (pg/mL) | **Healthy**  **control**  (*n* = 3)  mean ± SD  (pg/mL) | | | **Dabigatran**  **vs.**  **Saline** | **Dabigatran**  **sham**  **vs.**  **Saline sham** |
| KC (IL-8) | 594 ± 523 | | 1057 ± 590 |  | 128 ± 58 | 144 ± 52 | 89 ± 12 | | | p < 0.01** | p = 0.57 |
| IL-1b | 1630 ± 1733 | | 2313 ± 1817 |  | 97 ± 119 | 87 ± 55 | 42 ± 10 | | | p = 0.09 | p = 0.55 |
| IL-6 | 137 ± 155 | | 269 ± 259 |  | 10 ± 7 | 5 ± 3 | 9 ± 7 | | | p < 0.05* | p = 0.17 |
| IFN-g | 408 ± 493 | | 371 ± 414 |  | OOR< | OOR< | OOR< | | | p = 0.85 | n.a |
| G-CSF | 1.76 ± 1.8 | | 1.6 ± 1.1 |  | 0.16 ± 0.17 | 0.10 ± 0.08 | 0.21 ± 0.27 | | | p = 0.77 | >0.99 |
| IL-10 | 927 ± 701 | | 975 ± 501 |  | 189 ± 127 | 89 ± 35 | 198 ± 141 | | | p = 0.50 | p = 0.07 |
| IL-17A | 40 ± 27 | | 37 ± 19 |  | 21 ± 4 | 19 ± 4 | 22 ± 4 | | | p = 0.88 | p = 0.86 |
| VEGF | 173 ± 68 | | 185 ± 62 |  | 97 ± 28 | 102 ± 51 | 76 ± 28 | | | p = 0.57 | p = 0.97 |
| RANTES (CCL5) | 210 ± 79 | | 154 ± 55 |  | 55 ± 14 | 79 ± 18 | 69 ± 6 | | | p = 0.08 | p < 0.03* |
| ICAM-1 (CD54) | 3518 ± 1808 | | 5027 ± 1457 |  | 1263 ± 796 | 1204 ± 396 | 1457 ± 158 | | | p < 0.04* | p = 0.75 |
| L-selectin (CD62L) | 5802 ± 3215 | | 8189 ± 2443 |  | 1960 ± 995 | 2358 ± 571 | 1731 ± 648 | | | p < 0.03* | p = 0.28 |
| P-selectin (CD62P) | 3275 ± 3542 | | 4104 ± 3575 |  | 489 ± 642 | 2686 ± 548 | 976 ± 426 | | | p = 0.60 | p < .0001** |
| TIMP-1 | 8095 ± 11186 | | 14619 ± 9026 |  | 2582 ± 3170 | 2488 ± 2359 | 604 ± 41 | | | p < 0.02* | p = 0.87 |
| vWF ^#^ | 1.1 ± 0.6 | | 1.0 ± 0.5 |  | 0.5 ± 0.2 | 0.6 ± 0.2 | 0.7 ± 0.1 | | | p = 0.72 | p = 0.67 |
| TAT | 224 ± 108 | | 207 ± 108 |  | 91 ± 20 | 62 ± 24 | 128 ± 48 | | | p = 0.77 | p = 0.05* |
| TF | 3620 ± 3516 | | 2439 ± 2280 |  | 342 ± 325 | 169 ± 116 | 526 ± 562 | | | p = 0.65 | p = 0.80 |
| KC/IL-10 ratio | 0.68 ± 0.31 | | 1.37 ± 0.88 |  | 0.79 ± 0.24 | 1.82 ± 0.93 | 0.58 ± 0.28 | | | p = 0.03* | p = 0.02* |
| KC/RANTES ratio | 2.81 ± 2.49 | | 9.34 ± 10.2 |  | 2.32 ± 0.88 | 1.94 ± 0.84 | 1.30 ± 0.20 | | | p = 0.02* | p = 0.99 |

All infected rats were treated with 20 mg/kg/day gentamicin.

P-values were calculated using Student’s unpaired t-test by logarithmic transformation of data. * p ≤ 0.05, ** p ≤ 0.01

^#^ Concentration in ng/mL

IE, infective endocarditis, KC, Keratinocyte-derived chemokine (rat analogue to human IL-8); IL, interleukin, VEGF,

vascular endothelial growth factor, ICAM-1, Intercellular adhesion molecule 1, TIMP-1, tissue inhibitor metallopeptidase 1,

L-selectin, cell adhesion molecule of activated leukocytes, cell adhesion molecule of activated platelets and endothelial cells,

vWF, von Willebrand Factor, TAT, thrombin-antithrombin complex, TF, tissue factor. SD, standard deviation,

ORR<, out of range below detection level.
