# Supplementary material for: Adjunctive dabigatran therapy improves outcome of experimental left-sided Staphylococcus aureus endocarditis
Source: PLoS One. 2019 Apr 19;14(4):e0215333. doi: 10.1371/journal.pone.0215333 (PMC6474597; doi:10.1371/journal.pone.0215333)
Supplement: S2 Table — (DOCX) [file pone.0215333.s006.docx]

| **S2 Table. Results of ROTEM performed in plasma samples** | | | | |
| --- | --- | --- | --- | --- |
|  | **INTEM** | | **EXTEM** | |
| **Group**  Parameters | **Dabigatran**  (mean ± SD) | **Saline**  (mean ± SD) | **Dabigatran**  (mean ± SD) | **Saline**  (mean ± SD) |
| CT (s) | 250 ± 182 | 150 ± 16^a^ | 32 ± 4 | 31 ± 42^a^ |
| CFT (s) | 140 ± 289 | 25 ± 8^a^ | 26 ± 23 | 19 ± 10 |
| Alpha angle ( ̊ ) | 76 ± 24 | 85 ± 2^a^ | 87 ± 1 | 87 ± 1 |
| MCF (mm) | 41 ± 10 | 42 ± 6^a^ | 44 ± 5 | 39 ± 6 |
| AUC (mm^2^) | 4071 ± 998 | 4137 ± 528^a^ | 4210 ± 1100 | 4233 ± 552 |

This table presents the results means ± standard derivation (SD) of INTEM and EXTEM analysis of fresh frozen plasma. CT, clotting time; CFT, clot formation time; MCF, maximum clot firmness; AUC, area under the curve.

^a^ One sample failed to give a valid result due to haemolysis of the sample.
