# Supplementary material for: Adjunctive dabigatran therapy improves outcome of experimental left-sided Staphylococcus aureus endocarditis
Source: PLoS One. 2019 Apr 19;14(4):e0215333. doi: 10.1371/journal.pone.0215333 (PMC6474597; doi:10.1371/journal.pone.0215333)
Supplement: S3 Table — (DOCX) [file pone.0215333.s007.docx]

| **S3 Table** | **Histopathological assessment** | | | | | | |
| --- | --- | --- | --- | --- | --- | --- | --- |
| **Groups** | **Neutrophil inflammation**  **adjacent to valves** | **Abscess in myocardium** | **Haemorrhage myocardium** | **Coagulation necrosis in myocardium** | **Kidney**  **inflammation** | **Kidney**  **neutrophil**  **infiltration** | **Kidney**  **necrosis^*^** |
| **Dabigatran group**  **(*n=*6)** | 3 +, 2 ++, 1 +++ | 5 n, 1 y | 5 n. 1 y | 3 n, 3 y | 1 n, 5 + | 5 -. 1 + | 1 -, 2 +, 3 ++ |
| **Saline group**  **(*n=*6)** | 3 +, 2 ++, 1 +++ | 6 n | 5 n, 1 y | 5 n, 1 y | 5 +, 1 ++ | 3-, 2+ | 1 -, 1 +,  3 ++, 1 +++ |
| **Untreated controls (*n*=5)** | 5 +++ | 4 n, 1 y | 5 n | 3 n, 2 y | 5 +++ | 5 +++ | 5 ++++ |

All rats had histopathological verified inflamed valve vegetations. *n*, number of animals;

n, no; y, yes; nil, (-); mild, (+); moderate, (++); severe, (+++) inflammation.

Kidney necrosis^*^ of 0%, (-); <5%, (+) 5-10%, (++); >15%, (+++), >50% (++++)
