# Supplementary material for: Adjunctive dabigatran therapy improves outcome of experimental left-sided Staphylococcus aureus endocarditis
Source: PLoS One. 2019 Apr 19;14(4):e0215333. doi: 10.1371/journal.pone.0215333 (PMC6474597; doi:10.1371/journal.pone.0215333)
Supplement: S1 File — (PDF) [file pone.0215333.s008.pdf]

Supplementary

Materials and Methods

Bacterial challenge and growth conditions

S. aureus (NCTC 8325-4), a laboratory derivative (cured of prophages) of the WT sepsis isolate 8325 was used in the present study. The isolate originates from a patient with *S. aureus* IE and expresses key virulence factors involved in IE including coagulase, clumping factors, α- and β-hemolysin, protein A. An overnight culture of *S. aureus* was grown in Luria-Bertani (LB) medium on a shaking table (37 ºC, 150 rpm). *S. aureus* (100 µL) were then regrown in fresh LB to logarithmic phase with optical density of 0.5 at 600 nm. *S. aureus* was centrifuged and washed in saline (0.9 %), and saline-diluted, to the desired inoculum of 0.5 x 10^7 colony-forming units (CFU) in 500 µL syringes and kept on ice until injection.

Dabigatran treatment

Dabigatran etexilate (Pradaxa, Boehringer Ingelheim, Basel, Switzerland) tablets of 75 mg were dissolved in 9 mL 0.9 % saline overnight at 37 ºC on shaking table (150rpm) and doses of 10 mg/kg in 300 µL syringe were administrated intraperitoneal (i.p.). Control rats received i.p. injections with 300 µL 0.9 % saline.

Study animals

Experimental endocarditis model

All animal experiments were approved by The Animal Ethics Council and The Animal Experiments Inspectorate in Denmark (License no. 2013-15-2934-00952). High-grade aortic valve catheter induced mechanical lesions were produced in male Wistar rats (weight 225-250g) (Janvier Labs, Rennes, France), as

previously described [1,2]. Twenty-four hours after induction of the valve lesion rats were inoculated by intravenous injection by 0.5 x 10^7 CFU of *S. aureus* in a tail vein. Catheters were removed immediately before inoculation of the bacteria simulating native valve endocarditis conditions. Sterile thrombotic endocarditis was produced in sham control rats by catheter induced mechanical lesions without injection of bacteria, as previously described [1,2]. Before catheter procedure all animals were anaesthetised with a mixture of Hypnorm (fentanyl 0.315 mg/mL and fluanisone 10 mg/mL), sterile water and midazolam (5 mg/mL) in 1:2:1 dilution. Postoperative all rats received buprenorphine s.c. (0.05 mg/kg) every 8h for 48h post procedure. All rats were maintained in specific-pathogen-free conditions, monitored at least 3 times per day and had free access to water and food ad libitum. Rats reaching an endpoint, suffering or distress were sacrificed with pentobarbital/lidocaine i.p.

Intervention groups

All rats with IE were treated with gentamicin 20 mg/kg/day (Hexamycin i.v. solution, Sandoz A/S, Denmark) subcutaneously (s.c.) initiated one day post-infection (DPI), as described previously [1,2]. Gentamicin monotherapy was designed to mimic conditions of suboptimal antibiotic therapy of left-sided *S. aureus* IE and to be able to evaluate the effect of the adjunctive therapy [2]. Rats treated with adjunctive dabigatran etexilate received a dose of 10 mg/kg in 300 µL saline solution i.p. at 12-hour intervals (b.i.d). Dabigatran etexilate is a prodrug, which is converted in mammalian tissue to the active form, dabigatran, acting as a direct inhibitor of thrombin. Throughout the experiments, rats were maintained in specific pathogen–free conditions and had free access to chow and water.

Infected rats with severe *S. aureus* IE were randomized into two intervention groups: 1) intervention receiving adjunctive dabigatran (10 mg/kg b.i.d.) and gentamicin (20 mg/kg/day) (dabigatran group, n = 12) and 2) intervention receiving saline and gentamicin (saline group, n = 11). Rats with catheter-induced valve lesions, but without inoculation of bacteria developed sterile thrombotic endocarditis. These rats were randomized into: 1) sham controls receiving dabigatran (n = 6) or 2) sham controls receiving saline only (n =

6). The sham control group received the same dose of dabigatran as used for infected rats. All rats were evaluated three days after bacterial inoculation (infected rats) or removal of catheter producing sterile thrombotic endocarditis (sham controls). Intervention groups received two days treatment before evaluation. An additional twelve rats, six in each group, with catheter-induced aortic *S. aureus* IE were randomized into the same intervention groups as mentioned above, for use in flow cytometry analysis of whole blood, ROTEM analysis and histopathology evaluation.

In vitro growth of *S. aureus* in plasma dabigatran

An overnight culture was diluted 100-fold in PBS and 10µL were added to plasma with different dabigatran concentrations (<25, 106, 544 and 730 µg/mL) measured in the pharmacokinetic study. Measurements were performed in duplicates over an 8-hour period measured by ELISA with optical density (OD), 595 nm.

Histopathology

For histopathological examination, rats with IE were used. Six allocated in dabigatran group, six in saline group, five untreated controls (progression reference group) and one healthy rat was used as background control. Dissected heart valves, myocardium, kidneys and spleen were removed *in toto* and immediately fixated in 10 % buffered formalin. Tissue were dehydrated, paraffin embedded and sectioned 5 µm thickness. Consecutive sections were stained with hematoxylin and eosin (H&E) and Martius Scarlet Blue (MSB) using standard protocols. The slides were evaluated by light microscopy by pathologist blinded to the treatment groups and digitally photographed with VisionTek digital microscope (Sakura Finetek). All organs were assessed by degree of inflammation, fibrin deposit in the vegetation, necrosis and haemorrhage. Immunohistochemical staining with CD61 (glycoprotein IIIa, Agilent Dako, Copenhagen, Denmark) was used to identify megakaryocytes in the spleen.

Flow cytometry and ROTEM analysis

Measurements of platelets, leukocytes and platelet-neutrophil complexes (PNC)

Reagents. The following fluorescence-labelled antibodies were used to separate panels. **Panel 1** (neutrophil panel) activated neutrophils CD11b (APC, BD Bioscience, San Jose, CA, US, 562102) and rat granulocytes HIS48 (FITC, BD, 554907). **Panel 2** (neutrophil-platelets complexes); resting platelets CD42d (glycoprotein V) (PerCP/Cy5.5, Nordic Biosite, Copenhagen, Denmark, 148508), activated platelets P-selectin (CD62p) (APC, Nordic Biosite, 148304) and HIS48 (FITC, BD, 554907).

Sample preparation. We established two assay panels, slightly modified, from Hurley and colleagues [3] and Peters and colleagues [4]. Briefly, 5 µl of each of the antibodies were prepared in tubes (BD Trucount™ tube, 340334) to measure absolute counts of leukocytes in **Panel 1**. Within 10 min from sacrifice 50 µL whole blood collected in blood tubes (BD vacutainer® Citrate tubes 4.5 mL, 3.8%, 367704) (Carefully handled to prevent platelet activation) were gently mixed with antibodies in Trucount tubes and incubated in the dark for 10 minutes at room temperature. Then 200 µl of red blood cell lysis buffer (BD FACS™ lysing Solution) were gentle resuspended for 10 minutes of incubation. Additional fixation of cells was done by adding 0.2 % paraformaldehyde in PBS, pH 7.4 and analyzed the same day. One control rat from each intervention group without antibodies was used as internal background controls. Pilot studies have shown that platelets become activated over time in blood tubes without any stimulation, why it is important to start the assay within 10 min from blood collection to prevent artificial platelet activation (data not shown).

Flow cytometer settings. All analyses were performed on FACSCanto™ flow cytometer (BD Biosciences, San Jose, CA, USA) with a 488-nm argon laser and a 633-nm He-Ne laser. Samples were analyzed at a low flow rate (10 µL/min) and ≥ 20,000 events were recorded per sample. Cytometer Setup and Tracking Beads (BD Biosciences, San Jose, CA, USA) were used for calibration of the instrument. Flow data were processed and analyzed by Flow Jo (v.10.0.8, LLC, Ashland, Oregon) and BD FACSDiva software v. 6 (BD Biosciences, San Jose, CA, USA).

ROTEM analysis. Fresh frozen plasma samples (-80℃) were used for rotational thromboelastometry (ROTEM) analysis on ROTEM® delta (Tem Systems Inc., Munich, Germany). The ROTEM INTEM test is initiated by activation of the intrinsic pathway by partial thromboplastin phospholipid and ellagic acid and ROTEM EXTEM test initiated by activation of the extrinsic pathway by recombinant tissue factor and phospholipids. INTEM and EXTEM of fresh frozen plasma are reproducible and comparable to whole blood ROTEM assessment in humans in means of coagulation time (CT), alpha angle (˚) and AUC [5]. All ROTEM assays were run for 60 min at 37 °C.

Pharmacokinetics of dabigatran. Fourteen healthy rats were used for evaluation of pharmacokinetics (PK) in rats injected i.p. with single dose dabigatran (10 mg/kg) (Pradaxa, Boehringer Ingelheim, Basel, Switzerland). Whole blood sampling (BD Citrate tubes, 367704) was performed from right atrium, under general anesthesia by lethal dose pentobarbital/lidocaine (1 mL i.p.). Two rats at each time-point (t=30, 60, 120, 180, 240, 300, 720 min) post-injection were used to measure plasma dabigatran (p-dabigatran) concentration. Subsequently, tubes were centrifuged at 2,500 g (RCF) for 15 min (room temperature). Supernatant were moved to Nunc™ tubes and stored at -20℃ and measured within one month. P-dabigatran were measured by standard turbidimetric method (ACL TOP 750, IL, Werfen, MA, USA)

Measurement of cytokine and cell integrins. A 9-plex magnetic beads assay (Bio-Rad, Hercules, CA, US) was used to measure the expression of the following cytokines and chemokines: granulocyte–colony-stimulating factor (G-CSF), keratinocyte-derived chemokine (KC, rat analogue to human IL-8), interferon (IFN)-γ, interleukin (IL)-1β, IL-6, IL-10, IL-17A, vascular endothelial growth factor (VEGF), regulated on activation, normal T Cell Expressed and secreted (RANTES, CCL5). A 3-plex magnetic bead assay (R&D systems, Abingdon, UK) measured surface integrin (glycoproteins) expression of intercellular adhesion molecule 1 (ICAM-1), metallopeptidase inhibitor 1 (TIMP-1), and leukocyte integrin L-selectin (CD62L). For multiplex analysis, LUMINEX® 200™ platform (Luminex Corporation, Austin, TX) (Software, Bio-Plex Manager™ 6.1) was used according to the manufacturer's instructions. P-selectin (EKR248), Tissue Factor (ERK2744), vWF (EKR758) and Thrombin-Antithrombin complex (EKR2730) (purchased at Nordic Biosite, Copenhagen, Denmark) were analyzed by ELISA.

Results

Histopathologic characteristics of tissue specific inflammation in *S. aureus* IE

To evaluate the systemic and organ specific manifestations, as well as the safety of dabigatran treatment histopathologic examinations were performed. All rats had histological verified IE with inflammation of the valve endothelium with attachment of vegetations. Positive staining of deposits of fibrin in vegetations were found in 4 out of 6 rats in both infected groups (**S1 A and S1 B Figs**). The underlying myocardial tissue in relation to the inflamed valves, were seen with severe inflammation (*n* =17) (**S3 Table**) (**S1 C and S1 D Figs**). The degree of inflammation, hemorrhage and necrosis of the myocardium, kidneys and spleen were comparable between the two intervention groups. In kidneys, there was a slight tendency of increased neutrophil inflammation in saline compared to dabigatran group. The spleens of both groups were seen with hyperplasia of the white and red pulp. The red pulp was predominated by neutrophil granulocytes, but an increased number of myeloid precursor cells and megakaryocytes were also seen (**S1 E and F Figs**), indicating increased extramedullary hematopoiesis compared to healthy controls. Necrosis of the kidneys (**S1 G and S1 H Figs**) was predominantly located in the renal cortex with random hit distribution in both kidneys. A tendency towards a more severe degree of necrosis was observed for the saline group compared to the dabigatran group. Overall, the histopathology evaluation between the two intervention groups showed no marked difference at time of evaluation. Importantly, no increased tissue bleeding was observed comparing the dabigatran treated to saline treated group. In comparison to the intervention groups, an additional group of untreated rats (*n* =5) was used as reference, showing severe acute inflammation in all tissues compartments. A high degree of necrotic and hemorrhagic tissue was seen in the kidneys (**S3 Table**).

Interaction between *S. aureus* and dabigatran in a plasma assay

To examine a potential dabigatran effect directly on the proliferation rate of *S. aureus* we incubated *S. aureus* at different plasma concentrations of dabigatran. At high plasma dabigatran concentrations (544, 740 µg/L)

the observed growth was delayed throughout the experiment (0 to 8 hours, $p < 0.0001$), indicating a direct anti-*S. aureus* effect of dabigatran in plasma (**S4 Fig**).

References

1. Lerche CJ, Christophersen LJ, Trøstrup H, Thomsen K, Jensen PØ, Hougen HP, et al. Low efficacy of tobramycin in experimental Staphylococcus aureus endocarditis. Eur J Clin Microbiol Infect Dis. 2015;34: 2349–2357. doi:10.1007/s10096-015-2488-5

2. Lerche CJ, Christophersen LJ, Kolpen M, Nielsen PR, Trøstrup H, Thomsen K, et al. Hyperbaric oxygen therapy augments tobramycin efficacy in experimental Staphylococcus aureus endocarditis. Int J Antimicrob Agents. Elsevier; 2017;50: 406–412. doi:10.1016/j.ijantimicag.2017.04.025

3. Hurley SM, Lutay N, Holmqvist B, Shannon O, Boer O de, Tanck M, et al. The Dynamics of Platelet Activation during the Progression of Streptococcal Sepsis. Cox D, editor. PLoS One. Public Library of Science; 2016;11: e0163531. doi:10.1371/journal.pone.0163531

4. Peters MJ, Heyderman RS, Hatch DJ, Klein NJ. Investigation of platelet-neutrophil interactions in whole blood by flow cytometry. J Immunol Methods. 1997;209: 125–35.

5. Schoergenhofer C, Buchtele N, Schwameis M, Bartko J, Jilma B, Jilma-Stohlawetz P. The use of frozen plasma samples in thromboelastometry. Clin Exp Med. Springer; 2017;17: 489–497. doi:10.1007/s10238-017-0454-5

Figure legends

S1 Fig. Histopathological representative illustrations of catheter induced *Staphylococcus aureus* (*S. aureus*) infective endocarditis (IE) (**A**) Valve vegetation of severe *S. aureus* IE by hematoxylin and eosin stain (H&E) (Magnification x10). (**B**) fibrin (red) sequestering *S. aureus* in valve vegetations (x10) Martius Scarlet Blue (MSB). (**C**) illustrating aortic root abscess in myocardium (x2.5) with (**D**) severe neutrophil inflammation and inflamed valve (x20). (**E**) spleen with hyperplasia of white pulp with (x20) (**F**) CD61 positive megakaryocytes (x20) (**G**) showing two large areas of necrosis in the renal cortex, one at each pole of the kidney, the biggest marked by a dark square (overview), (**H**) enlarged picture of the area marked by the dark square in picture (**G**) showing the characteristic infarction with tissue necrosis in the kidney (x2.5).

S2 Fig. Histopathological illustrations from rats in the saline group. (**A**) Severe neutrophil infiltration of the aortic valve and subendothelial (hematoxylin and eosin (H&E), magnification x20) and (**B**) counterstained with Martius Scarlet Blue (MSB) where the collagen is strongly blue but no stains for fresh (yellow) or mature (red) fibrin. (**C**) Inflamed spleen (H&E, magnification x40) and (**D**) counterstained with myeloperoxidase (MPO), magnification x40) displaying a high intracellular expression of MPO in the neutrophils.

S3 Fig. Flow cytometry of whole blood. Platelet-neutrophil complexes (PNC) **(A)**, neutrophils **(B)** and total leukocyte count (TLC) **(C)** are shown for the two intervention groups. Horizontal lines represent means ± standard derivation. * indicate $p < 0.05$. n.s., non-significant.

S4 Fig. Dabigatran inhibits growth of *S. aureus* in plasma. Growth curve of *S. aureus* incubated in plasma from rats at different dabigatran concentrations (25-730 µg/L) showing a delayed growth rate of *S. aureus* at high plasma dabigatran concentrations (544 and 730 µg/L), indicating a direct anti-*S. aureus* effect of dabigatran in plasma. $P < 0.0001$ at plasma dabigatran concentrations at 730 and 544 µg/L from 1-8 hours compared to < 25 µg/L plasma dabigatran. Experiments were performed in duplicates. Symbols and error bars indicating mean ± SEM.

S1 Table. Cytokines and adhesion molecules expression in aortic valve endocarditis.

S2 Table. Results of ROTEM performed in plasma samples.

S3 Table. Histopathological assessment.

S1 Table. Cytokines and adhesion molecules expression in aortic valve endocarditis

Cytokines and adhesion molecules (glycoproteins)	*S. aureus* IE: Dabigatran (*n* = 12) mean ± SD (pg/mL)	*S. aureus* IE: Saline (*n* = 11) mean ± SD (pg/mL)	Sterile endocarditis: Dabigatran sham (*n* = 6) mean ± SD (pg/mL)	Sterile endocarditis: Saline sham (*n* = 6) mean ± SD (pg/mL)	Healthy control (*n* = 3) mean ± SD (pg/mL)	Dabigatran vs. Saline	Dabigatran sham vs. Saline sham
**KC (IL-8)**	594 ± 523	1057 ± 590	128 ± 58	144 ± 52	89 ± 12	p < 0.01**	p = 0.57
**IL-1b**	1630 ± 1733	2313 ± 1817	97 ± 119	87 ± 55	42 ± 10	p = 0.09	p = 0.55
**IL-6**	137 ± 155	269 ± 259	10 ± 7	5 ± 3	9 ± 7	p < 0.05*	p = 0.17
**IFN-g**	408 ± 493	371 ± 414	OOR<	OOR<	OOR<	p = 0.85	n.a
**G-CSF**	1.76 ± 1.8	1.6 ± 1.1	0.16 ± 0.17	0.10 ± 0.08	0.21 ± 0.27	p = 0.77	>0.99
**IL-10**	927 ± 701	975 ± 501	189 ± 127	89 ± 35	198 ± 141	p = 0.50	p = 0.07
**IL-17A**	40 ± 27	37 ± 19	21 ± 4	19 ± 4	22 ± 4	p = 0.88	p = 0.86
**VEGF**	173 ± 68	185 ± 62	97 ± 28	102 ± 51	76 ± 28	p = 0.57	p = 0.97
**RANTES (CCL5)**	210 ± 79	154 ± 55	55 ± 14	79 ± 18	69 ± 6	p = 0.08	p < 0.03*
**ICAM-1 (CD54)**	3518 ± 1808	5027 ± 1457	1263 ± 796	1204 ± 396	1457 ± 158	p < 0.04*	p = 0.75
**L-selectin (CD62L)**	5802 ± 3215	8189 ± 2443	1960 ± 995	2358 ± 571	1731 ± 648	p < 0.03*	p = 0.28
**P-selectin (CD62P)**	3275 ± 3542	4104 ± 3575	489 ± 642	2686 ± 548	976 ± 426	p = 0.60	p < .0001**
**TIMP-1**	8095 ± 11186	14619 ± 9026	2582 ± 3170	2488 ± 2359	604 ± 41	p < 0.02*	p = 0.87
**vWF** [#]	1.1 ± 0.6	1.0 ± 0.5	0.5 ± 0.2	0.6 ± 0.2	0.7 ± 0.1	p = 0.72	p = 0.67
**TAT**	224 ± 108	207 ± 108	91 ± 20	62 ± 24	128 ± 48	p = 0.77	p = 0.05*
**TF**	3620 ± 3516	2439 ± 2280	342 ± 325	169 ± 116	526 ± 562	p = 0.65	p = 0.80
**KC/IL-10 ratio**	0.68 ± 0.31	1.37 ± 0.88	0.79 ± 0.24	1.82 ± 0.93	0.58 ± 0.28	p = 0.03*	p = 0.02*
**KC/RANTES ratio**	2.81 ± 2.49	9.34 ± 10.2	2.32 ± 0.88	1.94 ± 0.84	1.30 ± 0.20	p = 0.02*	p = 0.99

All infected rats were treated with 20 mg/kg/day gentamicin.
P-values were calculated using Student's unpaired t-test by logarithmic transformation of data. * p ≤ 0.05, ** p ≤ 0.01
[#] Concentration in ng/mL
IE, infective endocarditis, KC, Keratinocyte-derived chemokine (rat analogue to human IL-8); IL, interleukin, VEGF, vascular endothelial growth factor, ICAM-1, Intercellular adhesion molecule 1, TIMP-1, tissue inhibitor metallopeptidase 1, L-selectin, cell adhesion molecule of activated leukocytes, cell adhesion molecule of activated platelets and endothelial cells, vWF, von Willebrand Factor, TAT, thrombin-antithrombin complex, TF, tissue factor. SD, standard deviation, ORR<, out of range below detection level.

S2 Table. Results of ROTEM performed in plasma samples

	INTEM		EXTEM	
**Group** Parameters	**Dabigatran** (mean ± SD)	**Saline** (mean ± SD)	**Dabigatran** (mean ± SD)	**Saline** (mean ± SD)
CT (s)	250 ± 182	150 ± 16[a]	32 ± 4	31 ± 42[a]
CFT (s)	140 ± 289	25 ± 8[a]	26 ± 23	19 ± 10
Alpha angle (˚)	76 ± 24	85 ± 2[a]	87 ± 1	87 ± 1
MCF (mm)	41 ± 10	42 ± 6[a]	44 ± 5	39 ± 6
AUC (mm^2)	4071 ± 998	4137 ± 528[a]	4210 ± 1100	4233 ± 552

This table presents the results means ± standard derivation (SD) of INTEM and EXTEM analysis of fresh frozen plasma. CT, clotting time; CFT, clot formation time; MCF, maximum clot firmness; AUC, area under the curve.
[a] One sample failed to give a valid result due to haemolysis of the sample.

S3 Table

Groups	Histopathological assessment						
	**Neutrophil inflammation adjacent to valves**	**Abscess in myocardium**	**Haemorrhage myocardium**	**Coagulation necrosis in myocardium**	**Kidney inflammation**	**Kidney neutrophil infiltration**	**Kidney necrosis***
**Dabigatran group (*n*=6)**	3 +, 2 ++, 1 +++	5 n, 1 y	5 n. 1 y	3 n, 3 y	1 n, 5 +	5 -. 1 +	1 -, 2 +, 3 ++
**Saline group (*n*=6)**	3 +, 2 ++, 1 +++	6 n	5 n, 1 y	5 n, 1 y	5 +, 1 ++	3-, 2+	1 -, 1 +, 3 ++, 1 +++
**Untreated controls (*n*=5)**	5 +++	4 n, 1 y	5 n	3 n, 2 y	5 +++	5 +++	5 ++++

All rats had histopathological verified inflamed valve vegetations. *n*, number of animals;
n, no; y, yes; nil, (-); mild, (+); moderate, (++); severe, (+++) inflammation.
Kidney necrosis* of 0%, (-); <5%, (+) 5-10%, (++); >15%, (+++), >50% (++++)
